# Ignoring versus updating in working memory reveal differential roles of attention and feature binding^☆^

**DOI:** 10.1016/j.cortex.2017.12.016

**Published:** 2018-10

**Authors:** Sean J. Fallon, Rozemarijn M. Mattiesing, Nina Dolfen, Sanjay G. Manohar, Masud Husain

**Affiliations:** a Department of Experimental Psychology, University of Oxford, Oxford, UK; b Nuffield Department of Clinical Neurosciences, John Radcliffe Hospital, Oxford, UK

**Keywords:** Working memory, Attention, Binding, Irrelevant information

## Abstract

Ignoring distracting information and updating current contents are essential components of working memory (WM). Yet, although both require controlling irrelevant information, it is unclear whether they have the same effects on recall and produce the same level of misbinding errors (incorrectly joining the features of different memoranda). Moreover, the likelihood of misbinding may be affected by the feature similarity between the items already encoded into memory and the information that has to be filtered out (ignored) or updated into memory. Here, we investigate these questions. Participants were sequentially presented with two pairs of arrows. The first pair of arrows always had to be encoded into memory, but the second pair either had to be ignored (ignore condition) or allowed to displace the previously encoded items (update condition). To investigate the effect of similarity on recall, we also varied, in a factorial manner, whether the items that had to be ignored or updated were presented in the same or different colours and/or same or different spatial locations to the original memoranda. By applying a computational model, we were able to quantify the levels of misbinding. Ignoring, but not updating, increased overall recall error as well as misbinding rates, even when accounting for the retention period. This indicates that not all manipulations of attention in WM are equal in terms of their effects on recall and misbinding. Misbinding rates in the ignore condition were affected by the colour and spatial congruence of relevant and irrelevant information to a greater extent than in the update condition. This finding suggests that attentional templates are used to evaluate relevant and irrelevant information in different ways during ignoring and updating. Together, the results suggest that differences between the two functions might occur due to higher levels of attentional compartmentalisation – or protection – during updating compared to ignoring.

## 1 Introduction

A fundamental tool that researchers have at their disposal to understand complex thought is to discover systematic patterns of errors in our perception of the world and to track the mental states that precipitate these misunderstandings. An important, systematic error in perception is the illusory conjunction, which occurs when the features that make up different objects are erroneously joined together. For example, after being presented with a red triangle and a blue square, the triangle may be phenomenologically perceived, or at least reported, as blue. The frequency of illusory conjunctions has often been found to increase when attention is diverted and has been integral to the development of theories on the necessity of attention for binding (Treisman, 1998, Treisman and Schmidt, 1982).

**Fig. 1:**
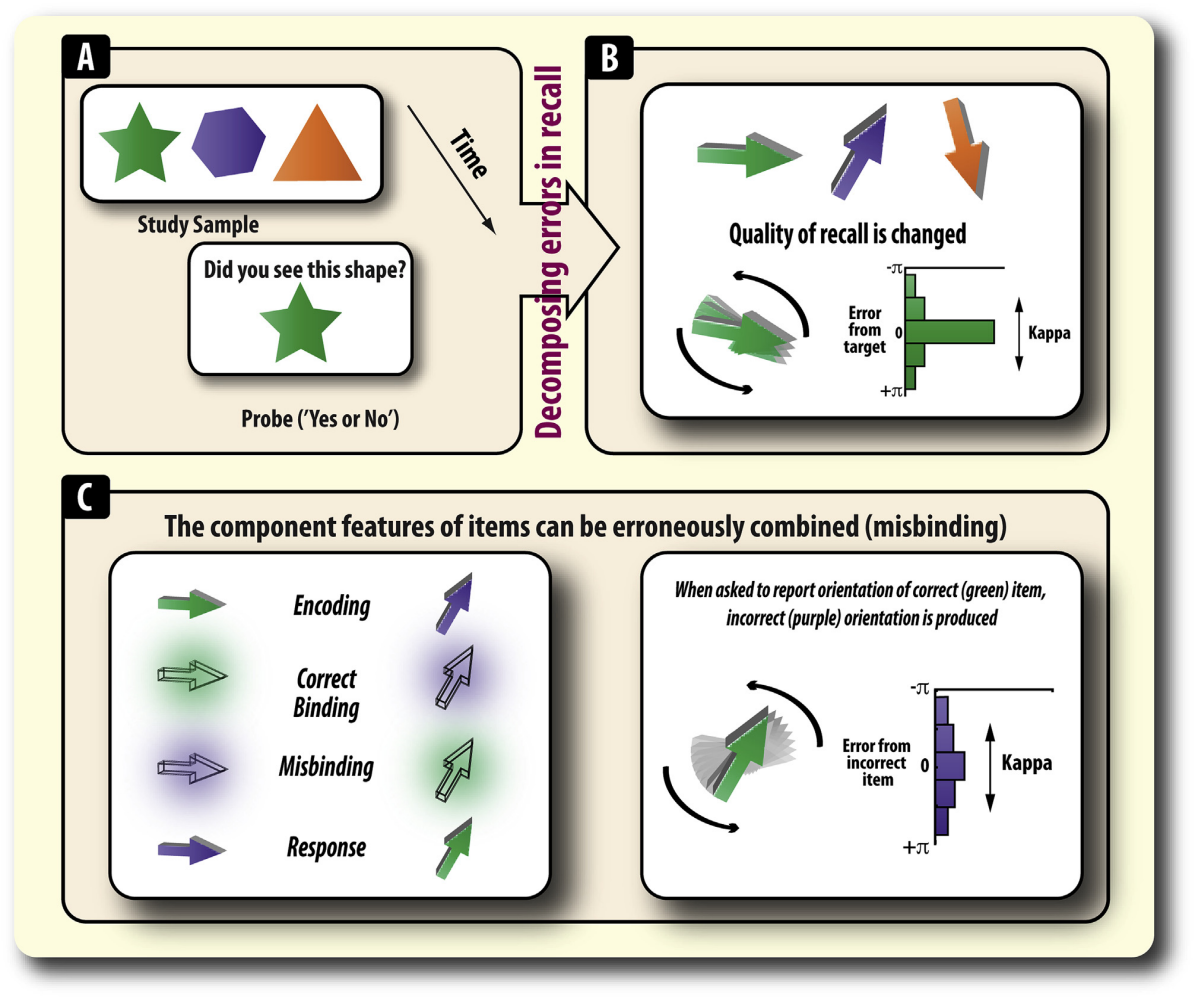
**Measuring recall on working memory tasks. A)** Traditional studies asked participants to make binary judgements on whether a probe item was one of the items they had to maintain. Thus, they provide an all-or-none view of WM recall. **B)** More recent methods probe WM by asking participants to reproduce the exact feature of a maintained item, e.g., its orientation, using a continuous, analogue – not binary – response space. This allows WM recall to be probed in a parametric fashion, e.g., measuring angular error. Therefore, the *quality* of recall can be assessed. By applying a stochastic model of WM recall (Bays et al., 2009), it is possible to decompose sources of error. Variability of recalling features of memoranda is captured in the model by the parameter [*kappa*]. Higher kappa values indicate lower variability (better recall) of retained items. **C)** When remembering several items, made up of different features, it is important that the right features (here colour and orientation) are correctly bound together. In Bays et al. (2009) model, the proportion of misbinding can be extracted. Finally, error can also occur due to random guesses (not shown, see text for details).

Similarly, the occurrence of illusory conjunctions may be critical to understanding the architecture of working memory (WM) and its relationship with attention. Although classic views of WM have argued that items are maintained as whole units (Luck & Vogel, 1997), recent research, in which participants are asked to reproduce the exact feature of a memorandum, has revealed that the orientation of encoded, but non-probed items, is sometimes reported instead of the probed orientation (Bays, 2015; Bays et al., 2009, Gorgoraptis et al., 2011, Schneegans and Bays, 2016). Such events are often referred to as misbinding or swap errors. Inclusion of misbinding errors has been found to be integral to understanding the nature of WM recall and form one of the key components in some computational models of WM (Fig. 1).

In recent years, researchers have uncovered a rich vein of reciprocal interactions between attention and WM (Braver and Cohen, 2000, Chun, 2011, Cowan, 2011, Engle, 2002, Gazzaley and Nobre, 2012, de Fockert et al., 2001). Attentional manipulations can affect misbinding rates in WM recall (Pertzov, Manohar, & Husain, 2017), suggesting that the deployment of attention during maintenance is crucial for understanding the genesis of these errors. However, it is unclear whether all types of attentional manipulations during WM maintenance have the same effect on the frequency of misbinding errors. Specifically, a key outstanding question is the extent to which ignoring sensory information while retaining information in WM and updating WM contents are two distinct operations and thus liable to produce different types of errors.

Arguing for a fundamental difference between these two functions is the finding that they recruit different nodes within the fronto-striatal network (Baier et al., 2010, Ekman et al., 2016, Fallon and Cools, 2014, McNab and Klingberg, 2008, Yu et al., 2013). However, both processes involve protecting WM recall from the influence of irrelevant information, suggesting that there may be some cognitive overlap. Confirming this idea, performance of both of these operations has been found to be improved by administering dopamine-enhancing medication to patients with Parkinson's disease (PD Fallon, Mattiesing, Muhammed, Manohar, & Husain, 2017). Thus, if the need to remove irrelevant information is the process responsible for generating misbinding errors, then *both* ignoring and updating should exert similar effects. In contrast, differential effects of ignoring and updating on misbinding would indicate that not all forms of attentional manipulation are equal in leading to corruption of information in WM. Demonstrating that one condition is more liable to induce greater levels of misbinding provides a means to probe the attentional mechanisms that are driving this effect.

**Fig. 2:**
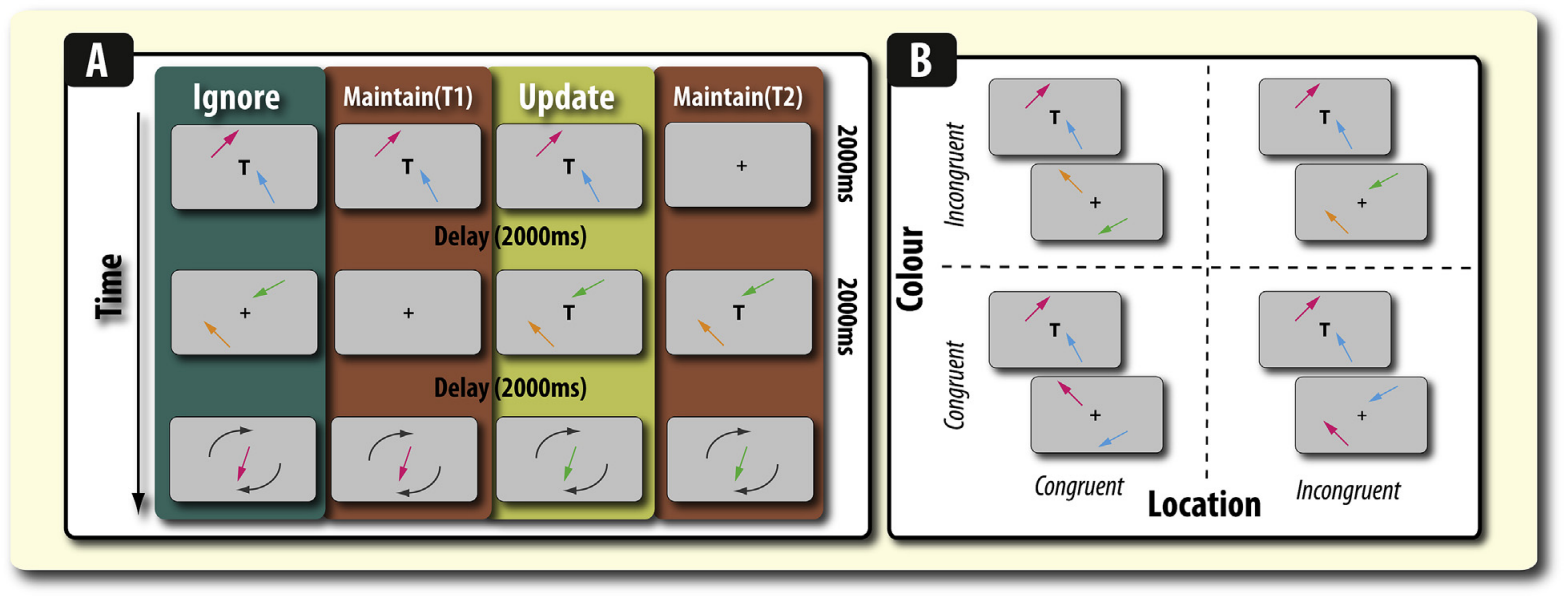
**Working memory paradigm A) Conditions.** A pair of coloured arrows (with different orientations) were presented for 2000 msec in all conditions. Across all trial types, WM recall error was measured by presenting a coloured arrow probe at the centre of the screen. Participants had to rotate this arrow so that it matched the target orientation. A key manipulation was to vary the presence of irrelevant information and the retention period for memoranda. The various trial types were randomly intermixed. One instruction served for all four trial types: Participants were instructed to remember *only* the most recently presented pair of arrows that had been presented with the letter “T”, which designated which pair of arrows were the arrows to-be-remembered (‘targets’). In the ignore condition (Far left), participants had to retain information whilst ignoring an irrelevant pair of arrows presented during maintenance. In contrast, in the update condition, participants were presented with two pairs of arrows consecutively, both of which were presented with the letter “T”. They had to remember the last pair of arrows, and discard – or jettison – the previous pair of arrows, which were now rendered irrelevant. Two temporal control conditions did not feature any irrelevant material but differed only in the length of time for which items needed to be retained. The maintain (T1) condition served as the temporal control for the ignore condition, whereas the maintain (T2) condition served as the temporal control for the update condition. **B) Four feature similarity variants.** The feature similarity between the targets and the irrelevant items in *both* the update and ignore conditions was varied by manipulating, in a factorial manner, whether they appeared in the same location and/or colour on the successive frames. For example, in the location congruent and colour congruent condition the targets and the distracters in the ignore condition both appeared in the same colour and in the same spatial location. Similarly, for the update condition, the first – subsequently irrelevant (‘ditched’) – pair of arrows appeared in the same colour and spatial location as the second pair of arrows.

Here, we examine the effect that ignoring and updating have on the fidelity of mental representations. We do this by employing a continuous report design whereby participants have to reproduce the exact orientation of memoranda. Participants were presented sequentially with two pairs of arrows, where either the first pair (update condition) or the second pair (ignore condition) were irrelevant at the point of recall (Fig. 2A). This allowed us to assess the overall quality of recall, while also being able to examine the specific pattern of errors in memory that each condition induces, i.e., whether both tasks induce similar levels of misbinding. Our previous study found that ignoring induced more misbinding errors in PD patients and healthy older adults (Fallon et al., 2017), but this question has not been fully explored in healthy young adults.

**Fig. 3:**
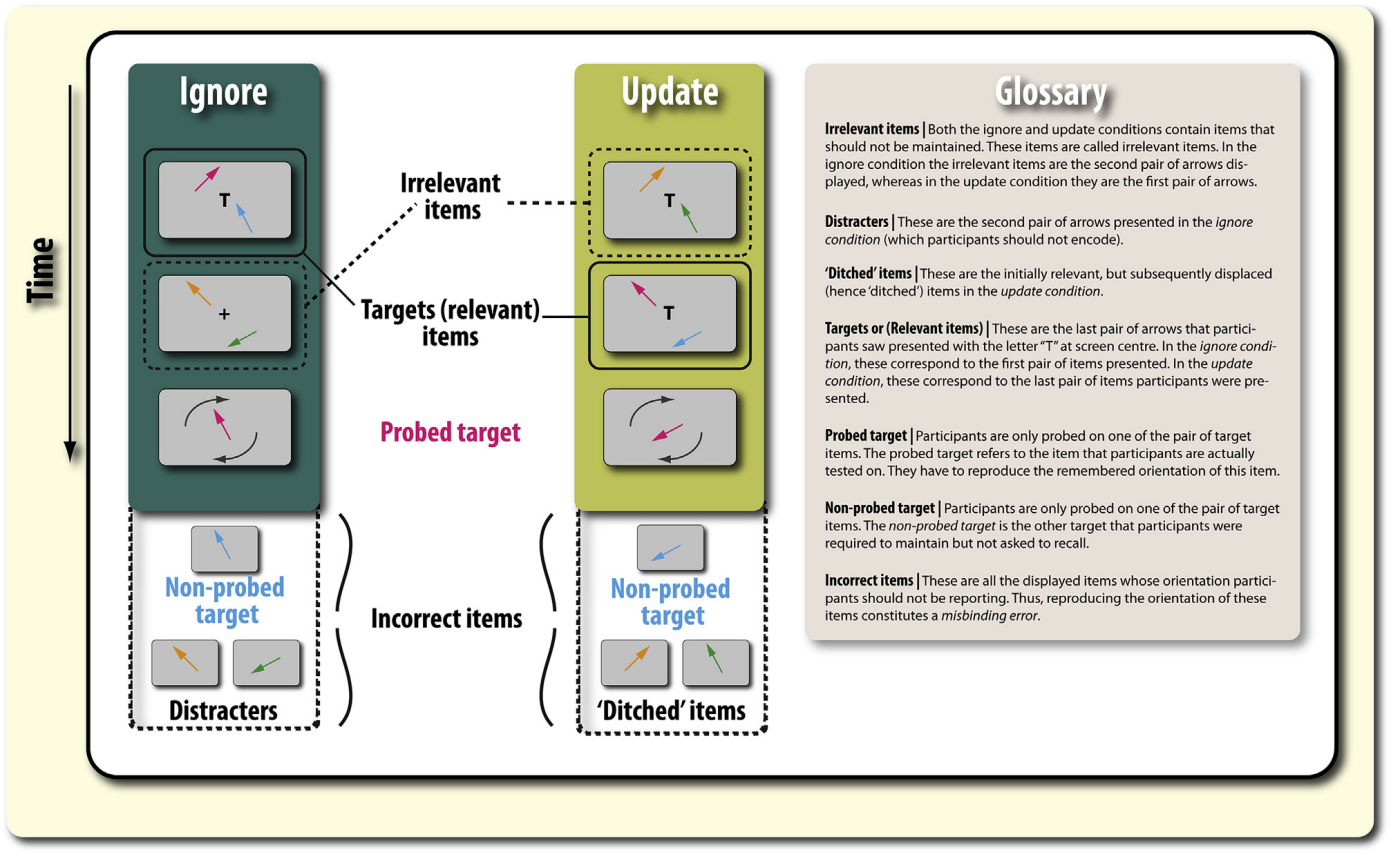
**Item misbinding types and glossary of terms.** Ignore and update trials are displayed (left). Both conditions require participants to maintain the orientation of two arrows (targets) and disregard the orientation of two irrelevant items. In the ignore condition, the irrelevant items are distracters that appear after the initially encoded memoranda. In contrast, in the update condition, the initially encoded memoranda are subsequently replaced by new targets. Thus, the initially encoded items are deemed irrelevant (‘ditched’). Participants are only probed (asked to report) the orientation of one arrow (the probed target). This leads to two types of incorrect items that participants could erroneously report (misbind) as the target item: the non-probed target and irrelevant items (which are, respectively, distracters and ditched items in the ignore and update conditions).

In addition, it may also be important to determine whether misbinding is induced by other items either previously seen (ignore condition) or held (update condition) in WM (Fig. 3). In most research on interference effects in WM, it is often tacitly assumed that irrelevant items can displace relevant information in WM. However, whether this actually occurs is rarely tested directly or quantified. Here, by applying a computational model of response selection that estimates the probability that participants were responding to a specific incorrect item (Bays et al., 2009, Schneegans and Bays, 2016), we attempted to determine whether any attentionally-demanding process (such as ignoring or updating) disrupts the maintenance of relevant information, or allows irrelevant information to completely displace relevant information.

A second question this study seeks to address, and one that was not explored in our previous report (Fallon et al., 2017), is to examine the modulatory role of feature similarity on the ability to ignore and update, particularly with respect to the levels of misbinding they induce. A fundamental neural mechanism of top-down control in WM is thought to be the increased activity that feature-specific cortical regions receive according to their task relevance (Miller and Cohen, 2001, Sreenivasan et al., 2014, Zanto et al., 2011, Zumer et al., 2014). Accordingly, due to competition for representation within corresponding cortical areas, distracters that are perceptually similar to the relevant information have been found to have a detrimental impact on WM (Jha et al., 2004, Yoon et al., 2006), and this effect has been found to vary with pharmacological modulation of top-down control areas (Bloemendaal et al., 2015). More directly, target and distracter similarity has previously been found to modulate misbinding rates during ignoring (Golomb, 2015). Similarly, updating WM with items more similar to the current memoranda has been found to have a facilitating effect (Ecker, Lewandowsky, & Oberauer, 2014). Thus, cognitively, ignoring and updating information might potentially be differentiated by the current contexts of WM in a task-specific manner.

One of the ways that the costs associated of dealing with attending to consecutively appearing visual presentations is to form an attentional template or ‘set’, whereby certain features or categories of information are excited or inhibited (Fallon et al., 2016, Olivers et al., 2006). The specific type of feature overlap – whether it is related to colour or location, for example – may be important in influencing attentional selection in WM. In order to manipulate the similarity between relevant and irrelevant information, we chose to examine the potentially independent effects of colour and spatial similarity by varying, in a factorial manner, whether the to-be-remembered and irrelevant items were presented in the same or different colours or spatial locations (Fig. 2B).

To summarize, although ignoring distracting information and updating current contents of WM both require dealing with irrelevant information it is unclear whether similar control mechanisms are associated with them. Here we investigate whether ignoring and updating induce the same level of corruption of memory by measuring three indices. First, we documented error in recall for these two conditions using a continuous, analogue report method. Second, we measured the level of misbinding at recall. Finally, because the likelihood of misbinding may be affected by feature similarity – between items already encoded into WM and information that has to be filtered out (ignored) or updated – we also investigated whether feature similarity differentially affects report in these two conditions.

## 2 Methods

### 2.1 Participants

Eighty-nine healthy young adults aged between 18 and 30 years of age (mean age = 22.38, SD = 3.10; 54 females and 35 males; see Supplemental Table 1) were recruited to take part in this experiment. Only participants with normal or corrected-to-normal vision and who were not colour blind were included in this study. All participants gave informed consent prior to participating in the study. Four participants were removed from the analysis due to having incomplete data sets. Two participants were subsequently removed from further analysis due to having extremely poor overall WM recall (>3 SD above overall mean for absolute mean angular error).

### 2.2 Design

This study used a delayed reproduction, or adjustment, task that allows the fidelity of WM representations to be assessed. The task required participants to encode the orientation of a pair of arrows, and then reproduce, after a delay, the orientation of one of the arrows. Thus, memory was not assessed in a binary fashion – remember or not – but in a continuous, parametric manner (Ma, Husain, & Bays, 2014).

This study comprised between (feature congruence) and within-subject (experimental condition) manipulations. The task featured four (intermixed) experimental conditions (Fig. 2A). 1) Ignore condition: after initially encoding items, distracters are presented. 2) Maintain (T1) – simple maintenance for same duration as the ignore condition (Ignore temporal control). 3) Update condition: after initially encoding stimuli, new stimuli were presented during maintenance that displaced the previous memoranda as items that had to be maintained. 4) Maintain (T2) – simple maintenance for same duration as the update condition (Update temporal control). Furthermore, the study contained four different feature congruency variants that varied the similarity between the relevant and irrelevant information in the ignore and update conditions (Fig. 2B). Due to the need to avoid over testing and inducing fatigue, separate groups of participants completed the four different feature congruency conditions (see below; Fig. 2B), while within each of these feature congruency conditions, participants completed all four experimental conditions (Fig. 2A). Note that an identical, but shortened, version of one of the four variants (Fig. 2B; colour incongruent location incongruent), was performed by PD patients and healthy controls in our previous study (Fallon et al., 2017).

In all conditions, participants saw two differently coloured arrows (randomly orientated) presented at different spatial locations equidistant from the centre of the screen for 2000 msec. At probe, they were shown only one arrow, with a randomly offset orientation, and were required to rotate the arrow until it matched the probed target orientation. For example, if a magenta arrow was presented at 45°, then, when presented with a magenta arrow at probe, participants had to rotate the probe arrow to 45°. After rotating the arrow to its desired orientation, participants had to press the space bar to confirm their response. Subsequently, they were given feedback about their performance-the orientations of the targets were simultaneously presented with their response.

Rather than being explicitly told to ignore or update items, the same instruction served to enable performance on all four tasks. Participants were simply instructed that they had to remember only the last pair of arrows that were presented with the letter “T” (displayed at the screen centre). This acted as a cue to instruct them that they should remember the arrows displayed on that screen.

**Ignore condition:** A pair of arrows was presented for 2000 msec with a “T” at screen centre, indicating that participants should remember these items. After a 2000 msec delay during which the screen was blank, another pair of arrows were presented (again for 2000 msec) with different orientations to those shown previously. The colour and location of these arrows differed according to experimental group (Fig. 2B; see below). This second pair of arrows (distracters) had to be ignored (signalled to the participant by the absence of the letter “T” and the presence of a fixation cross at screen centre). Participants' memory for target items was probed by being asked to reproduce the orientation of one of the target (to-be-remembered) arrows, indicated by colour. The probed target's colour was randomly drawn from one of the *first* pair of arrows seen on that trial. Thus, in this condition, there was a 6 sec delay between seeing the target items and being probed on one of them.

**Update condition:** Just as in the ignore condition, participants were presented with two sequentially presented pairs of arrows. But in this condition, both pairs had to be successively encoded as targets, because they were both accompanied by a central letter “T”. In this condition, the probed arrow came from the most recently seen (second) pair of arrows. Thus, the first set of arrows were rendered irrelevant (‘ditched’; Fig. 3) by the appearance of a “T” with the second set of arrows. In this condition, there was a 2 sec delay between the presentation of the target item and being probed on one of them.

The existence of a temporal disparity between the retention periods for the ignore and update conditions necessitated two separate temporal control conditions. The ignore and update conditions both had their own temporal control conditions in which no irrelevant information had to be removed (update) or prevented from entering (ignore) WM. Each of these was calibrated to have exactly the same retention period to match either the ignore or update condition. One maintain only condition served as a temporal control for the ignore trials (Maintain T1 in Fig. 2A). This condition was exactly the same as the ignore condition except that no distracters were presented during the delay period. Another maintain condition (Maintain T2 in Fig. 2A) acted as the control for the update condition. This was exactly the same as the update condition except that no targets were presented in the first frame. The ignoring and updating trials were identical in every single way, they differed only in the “T” on the first or second stimuli presentation (Fig. 2A).

**Fig. 4:**
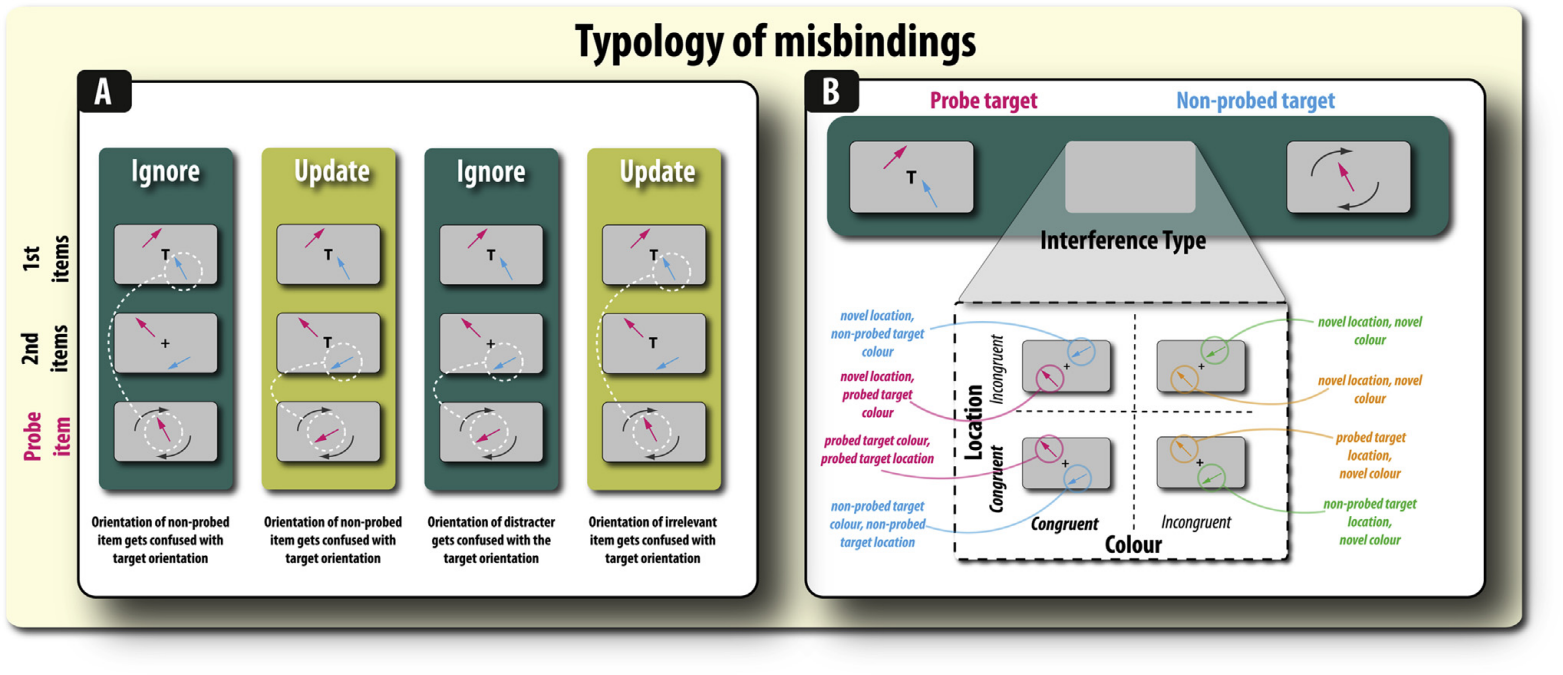
**Types of misbinding error. A) Types of misbinding in ignore and update trials.** In all four trial examples, the probed item is a magenta arrow. In the ignore and update conditions there are two types of misbinding that can occur. Misbinding can be to the other target item that was not probed (non-probed target) or it can be to one of the irrelevant items. In both the ignore and update conditions, misbinding to the non-probed target means that when probed on the target orientation the participant responded with the orientation of the item that appeared at the same time as the target. In the ignore condition, misbinding to the non-probed target is presented in the first frame (1st pair of items) and in the update condition, the non-probed target appears in the second frame (2nd pair of items). In both the ignore and update conditions, misbinding to the distracters means that the participant reproduces the orientation of one of the irrelevant items. In the ignore condition, the irrelevant items are the distracters that appear in the second frame. However, in the update condition, the irrelevant items are the initially presented (jettisoned) items presented in the first frame. B) Types of misbinding afforded by manipulating feature similarity. Manipulation of feature similarity means that there are further subtypes of misbinding that can occur depending on whether the colour or location of the irrelevant items is congruent with the target (to-be-remembered) items. Here, the variants of a ignore trial are depicted according to feature similarity.

Given that the effects of ignoring or updating may vary according to the overlap between the colours and spatial positions on the successive frames, we also examined, in a between-subject manner (i.e., across groups), the influence of varying these properties in a factorial manner (Fig. 2B). One group was exposed to trials where *both* the colours and spatial locations of the arrows (across frames) were kept *constant*. For a second group the *colours* of the arrows (across frames) were *different* but their spatial *locations were constant*. A third group was exposed to *same arrow colours*, but their spatial *locations varied*. Finally, for the fourth set of participants *both* the colours and spatial locations of the arrows *varied* across frames. This manipulation of feature similarity led to the possibility of different types of misbinding error (Fig. 4). Note that this manipulation was made across groups because of the need to maintain sufficient numbers of trials for each variation, given that we also had four main conditions (ignore, update and their two temporal controls, T1 and T2 respectively). Participants completed one session that contained 256 trials, thus there were 64 of each trial type.

### 2.3 Recall analysis

Mean angular error – the absolute angular difference between the target orientation (the orientation of the probed arrow) and the response orientation –was our main metric of recall. Data were analysed in SPSS 22.0 (IBM Corp). Mixed effects ANOVA was used to examine the effect of the within-subject factors retention period (2 *vs* 6 sec delay) and presence of irrelevant information (maintain *vs* ignore/update trials) and the between-subject factors colour congruence and location congruence. In the above model, a differential effect of ignore and updating, after correcting for time, would manifest as a significant interaction between retention interval (short and long) versus presence of irrelevant information (maintain only or irrelevant information present).

### 2.4 Modelling

Although mean angular error provides an indication of the overall level of WM recall, it is essentially a composite measure made up of potentially many different factors (Fig. 1). The exact sources of error in WM recall, and the possible mental representations that give rise to them, may be uncovered by examining the *pattern* of errors according to task (ignore or update) and feature similarity. A mixture model (Bays et al., 2009, Schneegans and Bays, 2016) has previously been used to uncover how dopamine and Parkinson's disease affect distinct aspect of WM recall (Fallon et al., 2017, Zokaei et al., 2014). This model sees WM recall as comprising four components, represented in the following equation:

$$p\left(\hat{\theta }\right)=\alpha \phi_{\kappa }\left(\hat{\theta }-\theta \right)+\sum_{i=1}^{m}\beta_{i}\phi_{\kappa }\left(\hat{\theta }-\varphi_{i}\right)+\gamma \frac{1}{2\pi }$$

1. **Variability in recall** (referred to as ***kappa*** or *κ*; Fig. 1C; **right**).
2. Probability of responding to the target orientation (α; Fig. 1C; **left**).
3. Probability of responding to incorrect items (β; Fig. 1D). The value of this parameter reflects the *probability* of misbinding and separate values can be obtained for each item type (Fig. 3, Fig. 4).
4. Probability of guessing (γ).

where $\hat{\theta }$ is the response angle, $p\left(\hat{\theta }\right)$ is the probability of the given response, $\phi_{\kappa }$ is a von Mises probability density function centered on zero with concentration *κ*, m is the number of incorrect items in the display (in this case 1 or 3, indexed by *i*), θ is the probed target angle, $\varphi_{i}$ are the angles of the incorrect items, and α, β_*i*_, γ are proportions of each component of the response distribution, satisfying $\alpha +\sum \beta_{i}+\gamma =1$. This model therefore has three free parameters, α,β,κ. Expectation maximization was used to obtain the maximum-likelihood-derived (Myung, 2003) parameters (see Bays et al., 2009 for details). The separate weights for each item were then classified according to the specified taxonomy (Fig. 4). The models were fit separately for each participant separately and each task and group analyses were performed on the extracted parameters. The model was found to be a good fit to the data (see Supplementary Materials).

### 2.5 Misbinding analysis

Our main interest in this study was to examine how misbinding varied according to task (ignore or update; Fig. 2A), feature similarity (of irrelevant items; Fig. 2B) and examining the pattern of error to specific items (Fig. 4A and B). Accordingly, we conducted four separate main analyses to examine distinct, but overlapping, questions. We examined:

A) ***Overall levels of misbinding*** to incorrect items according to retention period and presence of irrelevant information (similar to that for overall WM recall error).
B) ***Relative pattern of misbinding to relevant and irrelevant items on ignore and update trials (irrespective of feature congruence)***. The relative pattern of intrusions between relevant non-targets and irrelevant items can provide another important window on the role of attentional mechanisms in supporting the binding of information in WM.
C) ***Misbinding to non-probed target:*** How the pattern of misbinding to the non-probed target varied according to retention period and presence of irrelevant information. This involved comparing misbinding in the ignore and update conditions with their temporal controls. This analysis was conducted to examine whether the effects of ignoring or updating on misbinding could simply be accounted for by the effects of retention period.
D) ***Relative binding to different distracter items according to their similarity with the probed target:*** In addition to the similarity between targets and irrelevant items in the ignore and update conditions, there is a further, finer-grained distinction that can be made. Although participants have to retain the information of two items over the course of a trial, they only have to recall *one* of these items. Thus, a further question we can ask concerns the extent to which misbinding is affected by the similarity between the actual probed target and the distracter item that have different orientations, but share the exact same features (colour and location), i.e., is the level of misbinding to the distracter item presented in the same colour and location as the *probed* item the same as the distracter item that did not share the same features as the probed item.

## 3 Results

### 3.1 Ignoring but not updating impairs recall

**Fig. 5:**
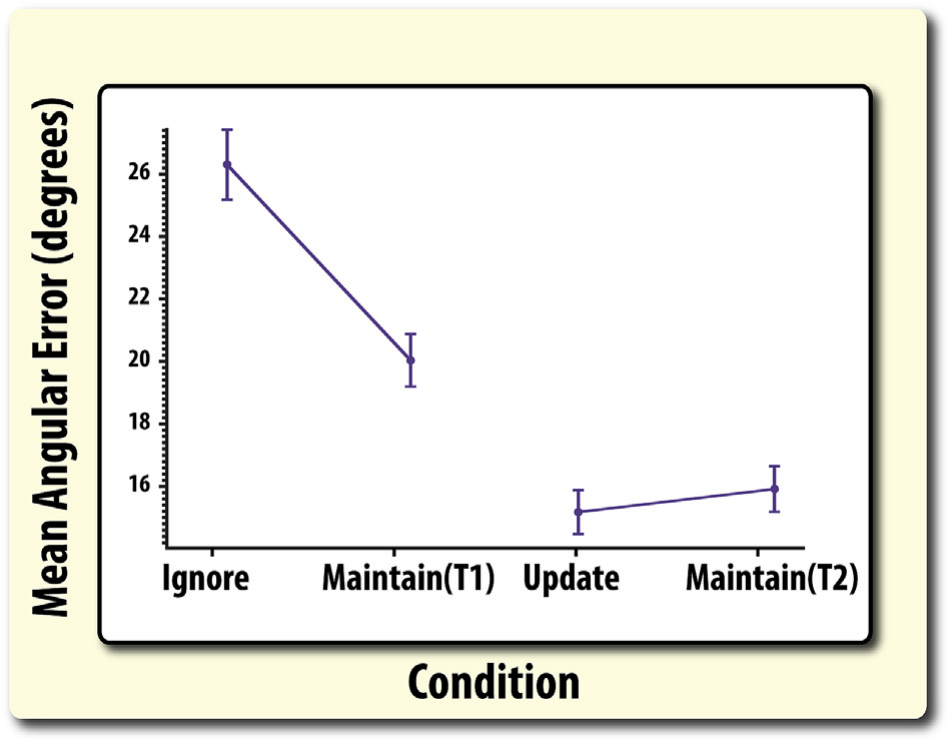
**WM recall (mean angular error) according to task.** Ignoring irrelevant information led to the greatest error in recall, even when comparing it to a matched unfilled retention duration (Maintain T1). Updating contents of WM led to no significant cost compared to its matched shorter temporal duration (Maintain T2). Error bars reflect standard error of the mean (SEM).

Longer retention periods produced significantly higher recall error (*F*(1,79) = 168.19, *p* ≤ .0001). Moreover, exposure to irrelevant information, compared to just maintaining, also significantly increased error (*F*(1,79) = 24.18, *p* < .0001; Fig. 5). Crucially, there was a significant interaction between retention period and type of irrelevant information (*F*(1,79) = 49.85, *p* < .0001). This was due to recall error on **ignore trials** being higher compared to its temporal control, T1 (*t*(79) = 7.53, *p* < .001). By contrast, this was not the case for the update condition compared to its shorter temporal control, T2 (*t*(79) = 1.11, *p* = .270).

There was no significant main effects of colour congruency (*F*(1,79) = 3.57, *p* = .062) or location congruency (*F*(1,79) = 3.56, *p* = .063), and none of the interactions were significant (*F*s < 1)*.* In summary, with regards to the overall quality of recall, ignoring exerted a disproportionate cost on recall, but updating did not. Moreover, colour and location congruency did not appear to affect WM recall significantly.

### 3.2 Decomposing the sources of error in WM recall

Next, in order to decompose the angular error into its component parts (i.e., the sources of error) we applied a probabilistic model of response selection, in which participants' errors are decomposed into four main components (see Methods and (Bays et al., 2009). Our main interest here was to examine whether task (ignore and update) and feature similarity affect misbinding levels. A misbinding event occurs when an orientation and a colour are erroneously combined (Fig. 1). As noted previously, there are also different types of misbinding participants can make (Fig. 4). The next sections correspond to examining the following four questions:

A) Overall misbinding across the four tasks (irrespective of feature congruence)
B) Differential misbinding to non-probed targets and irrelevant items (irrespective of feature congruence).
C) Misbinding to the non-probed relevant item in ignore and update conditions compared to their temporal controls
D) Relative binding to different distracter items according to their similarity with the probed target.

A) Ignoring disproportionately increases misbinding in recall

**Fig. 6:**
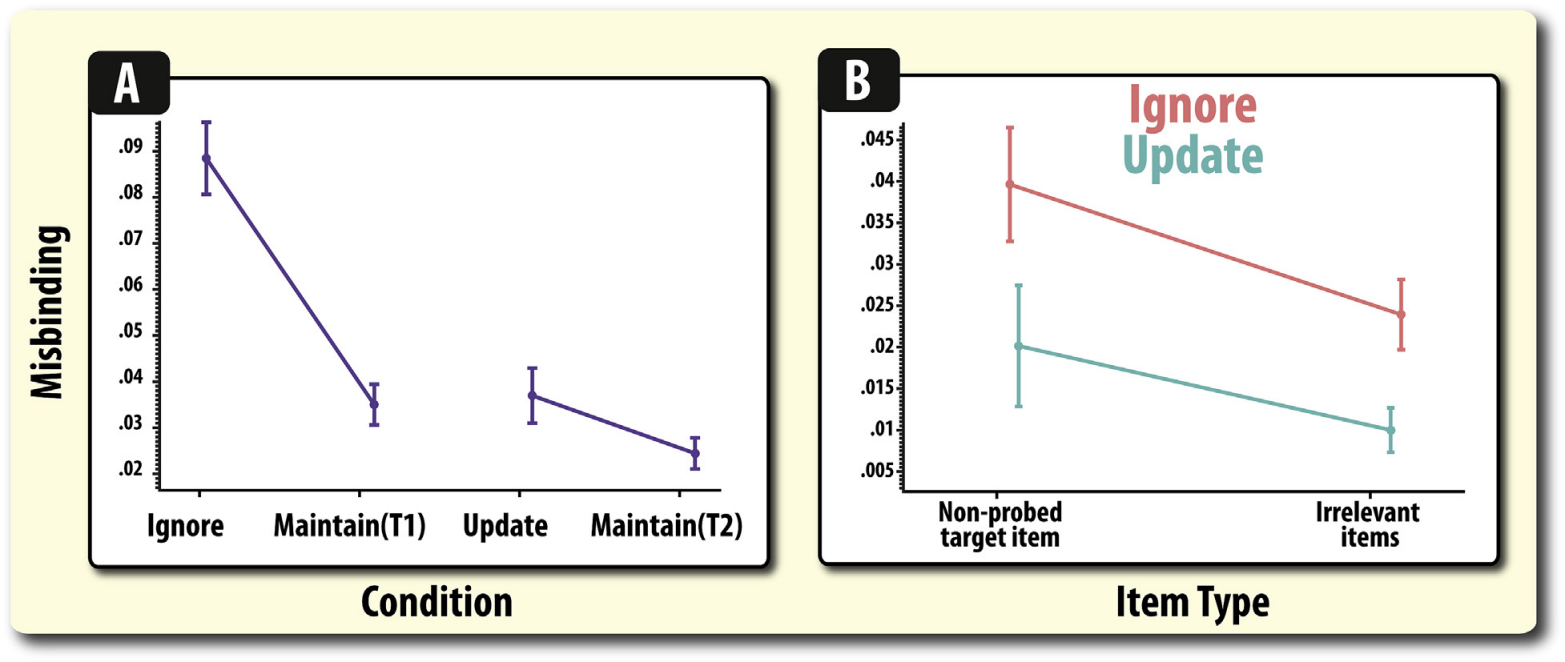
**Total misbinding according to condition and by item type. A)** Ignoring irrelevant information disproportionately increased misbinding, both compared to its temporal control (Maintain T1) and when the number of presented items was matched (update condition). Note, misbinding here is *total* misbinding to all incorrect items (Fig. 3). **B)** Misbinding in the ignore and update conditions according split according to the type of item that participants were misreporting (misbinding). Note, misbinding has been averaged across both irrelevant items. In both graphs, error bars reflect standard error of the mean (SEM).

Both retention period duration (*F*(1,82) = 35.92, *p* < .001) and the presence of irrelevant information increased misbinding (*F*(1,82) = 44.24, *p* < .001; Fig. 6A). Importantly, there was also a significant interaction between these two factors (*F*(1,82) = 16.52, *p* < .001). This was specifically due to increased misbinding in the ignore condition compared to its temporal control (*t*(82) = 6.62, *p* < .001), but not for the update condition (*t*(82) = 2.00, *p* = .062). This result echoes the pattern established for gross angular error (Fig. 5).

B) Disproportionate increase in misbinding to non-probed targets compared to irrelevant items in the ignore condition

The next question we asked was whether participants were more likely to report the orientations of either the non-probed target item (the other arrow that had to be remembered) or the irrelevant items, i.e., misbind, differentially in the ignore and update conditions, irrespective of feature congruence. Extracted estimates of misbinding were analysed in a repeated measures ANOVA with task (ignore or update) and item type (non-probed target, irrelevant item) as within-subject factors. Note, that the misbinding rates for the pair of irrelevant items in the ignore and update conditions were averaged.

As above, participants were significantly more likely to misbind in the ignore, compared to the update, condition (*F*(1,82) = 35.12, *p* < .001; Fig. 6B). There was a significant main effect of type (*F*(1,82) = 34.21, *p* < .001), with higher levels of misbinding to the non-probed target compared to irrelevant items. However, this was found to vary significantly according to task (*F*(1,82) = 17.71, *p* < .001; Fig. 6B). Misbinding was significantly higher to non-probed targets than distracters in the ignore condition (*t*(82) = 5.89, *p* < .001), whereas this effect was not significantly present in the update condition(*t*(82) = 1.81, *p* = .074).

C) Spatial and colour congruency affect feature misbinding

Next, we examined the effect of task (ignore or update) and feature similarity (location or colour) on misbinding. In this section, we restrict ourselves to examining the level of misbinding to the other arrow that had to be remembered (i.e., the non-probed target) in the ignore and update conditions versus their temporal controls. This analysis is important to exclude the possibility that the differences in retention period are driving the differential misbinding rates in the ignore and update conditions.

We performed an ANOVA that examined all four task conditions (ignore, maintain T1, update and maintain T2) and feature similarity conditions (location, colour) in influencing the level of misbinding towards the *relevant but non-probed target*. There was also a significant main effect of retention period, whereby longer retention periods were associated with increased misbinding (*F*(1,79) = 33.5, *p* < .0001). Although the presence of irrelevant information per se did not affect misbinding (*F* < 1), there was a significant interaction between retention period and the presence of irrelevant information (*F*(1, 79) = 5.65, *p* = .020). This significant interaction was due to misbinding to the non-probed target being *lower* in the update condition compared to its temporal control (*t*(79) = 2.87, *p* = .005), but with no such effect apparent for the ignore condition (*t*(79) = .94, *p* = .346).

**Fig. 7:**
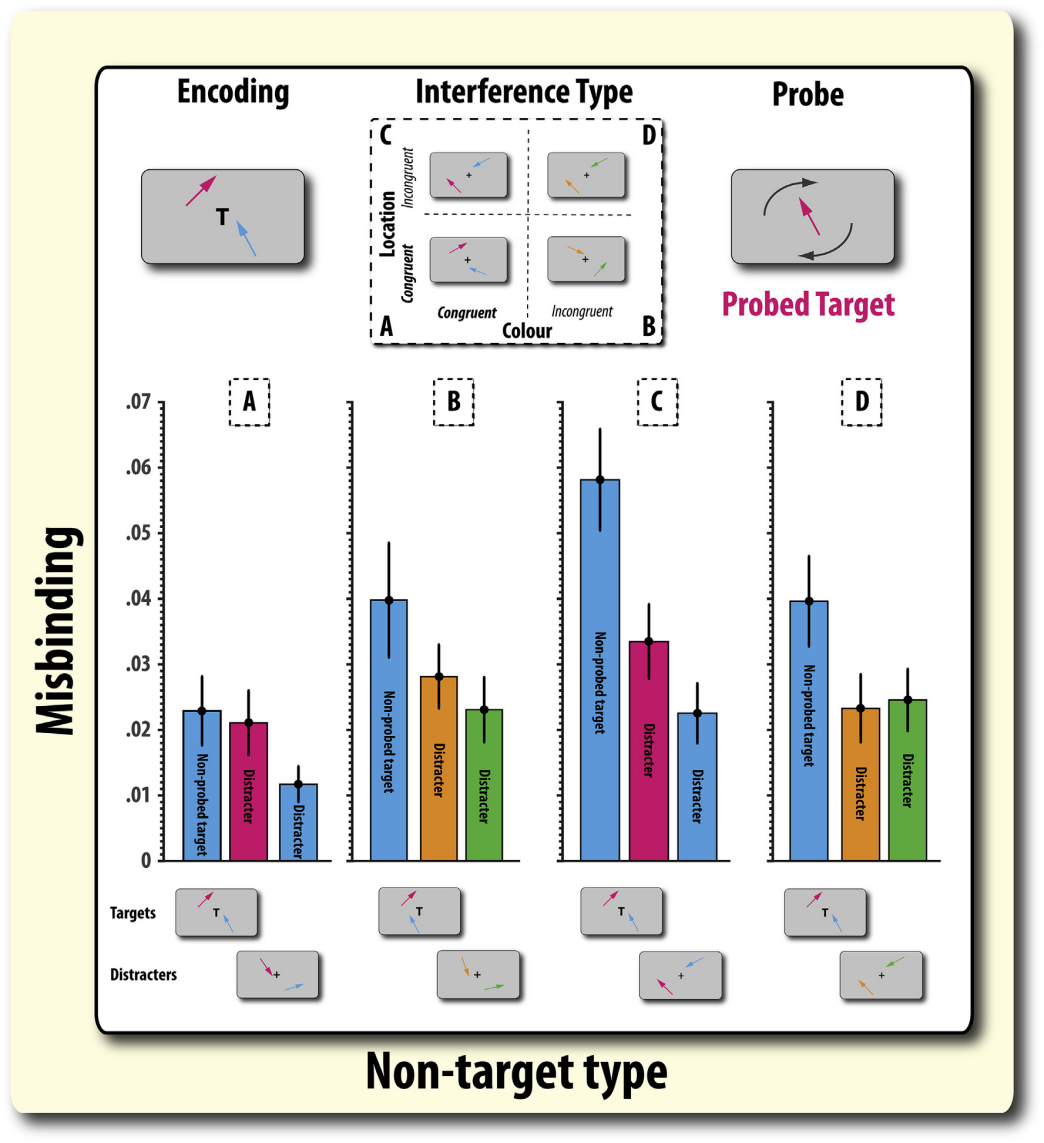
**Misbinding in the ignore condition according to the type of incorrect item and feature similarity condition. A–D:** Probability of misbinding: reporting the orientation of either a non-probed (incorrect) item or an irrelevant item (distracter). The likelihood of reporting the orientation of the non-probed target varied with the type of interference – colour and/or location congruence of irrelevant items. For more information on the type of incorrect item see Fig. 4A and B. Error bars reflect the standard error of the mean (SEM).

There was a borderline significant four-way interaction between retention period, presence of irrelevant information, colour congruence and spatial congruence (*F*(1,79) = 3.959, *p* = .05). Given the focus on understanding differential levels induced by ignoring and updating, we unpacked this interaction to see if there were separate interactions in the two conditions. There was no such three-way significant interaction between colour and spatial congruency on misbinding to the non-probed target in the update condition (t < 1). However, colour and spatial congruency did significantly interact to influence the level of misbinding to the non-probed target in the ignore condition (*F*(1,79) = 6.85, *p* = .011; Fig. 7; Supplemental Fig. 5). This interaction was due to misbinding being higher in the location incongruent than congruent condition when colour was congruent (*t*(79) = 3.80, *p* = .0003), but not when colours were incongruent (*t* < 1). Thus, in the ignore condition, having distracters appear in the same colours but different locations as targets was associated with an increase in misbinding compared to when these items appeared in the same colours but also the same locations. In contrast, no such effects were found in the update condition.

D) Misbinding occurs more to the distracter item that was presented in the same colour and location as the probed item

The final question we address is how the level of misbinding to each irrelevant item varies according to their colour and spatial congruence with the *probed* item (Fig. 7). Note that if reporting the orientation of distracters (misbinding) is higher when these items appeared in the same colour and spatial location *as the probed item*, then this effect would suggest that some misbinding occurs at retrieval because the probed item is not known until that point in time.

We performed a separate ANOVA to examine the relative pattern of misbinding to either the non-probed target or the irrelevant items, in the ignore and update conditions. Thus, we analysed which of the incorrect items' orientations was more likely to be erroneously reported as the probed target orientation: whether it was the non-probed target (the other arrow that had to be remembered) or irrelevant items that appeared in the same colour or location as the probed target or irrelevant items that appeared in the same colour or location as the non-probed target (Fig. 4B). Note that in the condition where targets and irrelevant items appeared in different colours and locations, irrelevant items were arbitrarily assigned to one of these categories for the purpose of analysis. Misbinding rates were examined in a mixed ANOVA with task (ignore or update) by type (non-probed target, congruent irrelevant item, incongruent irrelevant item) as within-subject factors and colour congruency (same or different colours for targets and irrelevant items) by location congruency (same or different location for targets and irrelevant items) as between-subject factors.

There was a four-way interaction between task (ignore, update), item type, colour congruency and location congruency (*F*(1, 158) = 5.08, *p* = .007; Supplemental Fig. 7). This interaction was due to there being a three-way interaction between type, colour-similarity and location-similarity for the ignore condition (*F*(2,158) = 3.36, *p* = .037; Fig. 7), but there was no such interaction for the update condition (*F*(2,158) = 1.68, *p* = .189). We next examined the crucial question of whether misbinding occurs more to the irrelevant item that matches the features (colour and location) of the probed item. Given that significant colour and location congruence effects were found only for the ignore condition, we restrict ourselves to examining this question for ignore trials.

The level of misbinding to the distracter item that was presented in the same colour and location as the probed item was significantly higher than to the distracter item that was not in the same colour or location as the probed item (*t*(20) = 2.56, *p* = .018; colour and location congruent; Fig. 7A). A non-significant trend was also observed for misbinding to the distracter item presented in the same colour as the probed item to be higher than the distracter item not presented in the same colour (*t*(20) = 2.07, *p* = .051; colour congruent, location incongruent condition; Fig. 7C). However, this was not the case in the other conditions (all *p*'s > .151; Fig. 7B, D). Thus, in conclusion, distracter items that are similar to the probed target exert a greater intrusive effect on recall. Therefore, this result reveals that some of the misbinding to distracter items is induced after or during the probe phase, i.e., at or after retrieval.

### 3.3 Other aspects of the model

Kappa – indicating the variability in which information was recalled – showed effects of retention period and varied by location and colour congruence. As this is not the main focus of our study, these effects are reported elsewhere (see Supplementary Material; Supplemental Figs. 1 and 2). Guess – the probability of reporting orientations unrelated to any of the presented items showed effects of retention period and the presence of irrelevant information (see Supplementary Material; Supplemental Fig. 4).

## 4 Discussion

The aim of this study was to examine the interaction of attention and WM when people either have to ignore irrelevant information – resist distraction – or update the contents of memory so that previously relevant items now become irrelevant. WM recall was significantly worse after ignoring compared to updating (Fig. 5). A specific focus of this research was to examine whether these two operations, which both require filtering out irrelevant information, induce the same levels of misbinding. Moreover, the feature similarity (colour and location) of relevant and irrelevant items was manipulated to expose whether common or task-specific effects of ignoring and updating on misbinding were observed. Our misbinding analyses revealed several facets about how WM recall goes awry in a systematic fashion according to the demands placed on the attentional system: presenting distracters (ignore condition) induced a higher level of misbinding than in the update condition, even after taking into account temporal differences (Fig. 6A). Moreover, increased misbinding in the ignore condition was also found to be due to an increase in misbinding to the non-probed target rather than the distracters themselves.

Expanding the results of our previous study (Fallon et al., 2017), the effects of feature similarity were also larger in the ignore compared to update condition, indicating the differential roles of attentional sets (excitation and inhibition of relevant and irrelevant features) in influencing misbinding rates in these conditions. Finally, reporting the orientation of distracters (misbinding to distracter items) was higher if the distracters appeared in the same colour and same spatial location *as the probed item*. This result reveals the potential importance of retrieval processes on misbinding. These changes in misbinding levels were observed despite overall recall error being equal across different feature congruency conditions. This makes it unlikely that the misbinding differences were driven by overall difficulty between the conditions or were driven by group differences between the participants who performed each feature congruence conditions.

Cumulatively, therefore, the results of the current study reveal that ignoring and updating appear to be dissociable processes in WM with different patterns of corruption of mental representations associated with them.

### 4.1 A specific kind of attention is necessary to maintain binding of relevant information

The occurrence of illusory conjunctions has played a key role in informing theories of attention (Treisman, 1998). Here, we employed similar logic to examine the types of systematic error that occur during WM recall as a function of task and feature similarity. Participants made systematic errors when recalling the orientation of a probed target item. Instead of veridically reproducing the orientation of the probed target item, they frequently reported the orientation of incorrect items instead, suggesting that the mapping between colour and orientation become displaced – or misbound – in WM.

An area of controversy in object perception research is whether attention is necessary to bind features together (Humphreys, 2016, Treisman, 1998, Wolfe, 2014). This issue has also transferred itself to the mnemonic domain. Some accounts of WM consider objects to be stored as unified items, with the number of features present within an object *not* impacting upon storage capacity (Luck & Vogel, 1997). In contrast, others report that binding items in WM is resource demanding and affected by the number of object features retained (Wheeler & Treisman, 2002). Binding of information can be corrupted by the presentation of subsequent stimuli (Allen, Baddeley, & Hitch, 2014). In addition, a recent study reported that retrocues – cues presented after encoding that indicate which items are going to be probed – decrease misbinding (Wildegger, Humphreys, & Nobre, 2016). These results suggest that attention is necessary to maintain the correct bindings between features as the presentation of distracters – which presumably consumes attentional resources – dramatically increased misbinding rates. However, prior to the present study, whether the putatively different forms of attentional manipulation of ignoring and updating information in WM exert the same effect on misbinding was not known. Despite the fact that they share the common feature of having to deal with irrelevant information, ignoring exerted a disproportionately greater increase on misbinding compared to updating. This strongly suggests that it is the distinct form of attentional engagement induced, or perhaps prevented, by ignoring that increases misbinding. It might be suspected that some form of perceptual decoupling or disengagement from processing external stimuli, i.e., mindwandering (Smallwood & Schooler, 2015) is responsible for inducing this effect. However, there is evidence for separate effects of ‘internal’ and ‘external’ distraction on cognitive performance (Chun, Golomb, & Turk-Browne, 2011). In addition, the susceptibility of ignoring to congruency effects (discussed below) strongly argues against this hypothesis.

The results suggest that there may be different attentional mechanisms involved in supporting ignoring and updating. Attending to items in WM is often thought to bring them into a privileged state or the focus of attention (FoA; Cowan, 2011, Souza and Oberauer, 2016). The demands of bringing an item into and out of the FoA may underlie differential misbinding rates. Keeping an item in the FoA has been shown to be an effective strategy for reducing the effects of distraction (Gorgoraptis et al., 2011). Thus, in our task, participants may be engaged in actively maintaining relevant items in the FoA while processing distracters in the ignore condition. Competition for entry into the FoA may lead to the corruption of information and misbinding. In line with this interpretation, distracter presentation not only abolishes certain patterns of neural activity but also generates others (Artchakov et al., 2009), suggesting that new, potentially corrupted, representations are formed after an ignoring event. A lack of attentional compartmentalisation (competition) between relevant and irrelevant information is supported by the finding in our study that misbinding varied according to the feature similarity of targets and distracters.

In contrast, the relatively reduced levels of misbinding in the update condition might be due to the lack of competition for entry into the FoA. In this condition, initially encoded information has to be fully removed (ditched) when a second pair of targets is presented. This manoeuvre potentially facilitates smooth removal of old information from WM and allows new items to enter with *reduced competition*.

### 4.2 Efficient selection between relevant information (targets) is modulated by colour and location congruence of irrelevant items

Our misbinding analysis identified that feature congruence effects were more prominent in the ignore compared to update condition. Participants were significantly more likely to report the orientation of the non-probed target (the other item they were supposed to retain in memory) when the targets and irrelevant items appeared in *different spatial locations* but in the *same colours* (Fig. 7C). Thus, colour congruence (targets and irrelevant items being presented in the same colour) caused location to have a bigger effect on misbinding level to relevant-but-not-probed items.

This result rules out the possibility that it is the degree of feature incongruence that determines the levels of misbinding errors participants make, i.e., that there is a linear effect of feature similarity on misbinding rates. Such an effect would result in a linear increase in misbinding across congruencies (Fig. 7 A–D). Thus, in contrast to studies that have reported that target-congruent distracters are always the most disruptive to WM (Yoon et al., 2006), our investigation did not find direct evidence for this claim on overall misbinding rates (though note, similarity may affect the probability of misreporting certain items, if not overall misbinding rates; see below). Rather, the results point to a synergistic (or asymmetrical) effect of spatial and feature information of distracters in the ignore condition corrupting the representation of relevant information.

There may be several factors that contribute to this synergy. However, the “preview” effect in visual search – whereby search for a target is augmented by pre-showing distracters in the same locations prior to search – suggests that visual discrepancies across frames can guide the selection of information (Watson, Humphreys, & Olivers, 2003). Analogously, a similar effect may occur here. It is possible that distracters that appear in the same colour in the same position may be perceived, to some extent, as the same item, increasing the efficacy with which these items are “tagged” as irrelevant, leading to greater inhibition of their representations and thereby induce less misbinding or corruption of relevant items (Fig. 7A). Conversely, having an item in the same colour appear in a new position may be perceived as the “same” object, but in a different, updated position and therefore worthy of attention, i.e., the appearance of targets in the same colours but at different locations with different orientations (as occurs in the colour congruent/location incongruent condition) could trigger an automatic “update” mechanism, whereby potentially important information about relevant information is deemed to have changed. This may increase the attention these items receive and thereby enhance misbinding. Possession of a mnemonic system which uses feature-based and location-based information in fundamentally different ways may be adaptive from an ecological perspective, where such detection of a predator or prey (same features) in a different spatial location may be salient and draw in attention. This view is also consistent with ideas that object-based and spatial WM can function relatively independently (Courtney et al., 1996, Darling et al., 2006, Owen et al., 1997, Postle et al., 1997). However, based on the present data we cannot fully discount an alternative suggestion that is in keeping with another model of WM (Oberauer, 2002), in which spatial and feature-based information exert different effects on misbinding due to the fact that they had differences in relevancy or due to the attention each dimension was given. Although the colour of the targets was relevant to eventual recall, the spatial location of the targets or distracters was irrelevant. Future studies will need to be conducted to adjudicate between these two hypotheses.

### 4.3 Intrusion of distracter items is affected by their similarity to the target items

WM involves the ability to efficiently maintain multiple items over a sustained period of time. In addition, another key component of WM is to allow accurate recall of information. It would be inefficient to have a rich neurocognitive architecture supporting the encoding and maintenance of items if those items could not be faithfully retrieved. This study's results suggest that some of the effects of feature similarity on ignore trials occur during the *retrieval* phase of WM. In all variants of this task, participants had to remember the orientation of two arrows but were probed on only one of these items. Thus, the identity of the probed item is, by definition, only revealed at the retrieval phase. If revealing the identity of the probed item had no influence on the pattern of misbinding, then rates of falsely reporting the orientation of both distracter items should be equivalent. However, this was not observed (Fig. 7A). Rather, this study found that misbinding rates were higher to distracters presented in the same colour and at the same location as the probed target item (compared to distracter item that matched the non-probed item). This suggests that some errors in WM recall occur only at the retrieval phase, and not at encoding or maintenance.

Several studies have shown that items stored in WM can capture attention in an automatic fashion (Olivers et al., 2006, Soto et al., 2005, Soto et al., 2008, Soto and Humphreys, 2009). The impact of WM-congruent distracters on underlying mental representations has had to be inferred based on reaction time data. Here, we were able to show that probed-target congruent distracters directly displaced target feature information (Fig. 7A). One possibility is that when presented with the probe item (drawn from the pair of target items), participants are mentally replaying the information they were presented with in order to retrieve the relevant information, and in the process, are encountering distracters that share feature information with the probed target. The neural mechanisms that support the temporal replay of information during the maintenance period in WM tasks have recently begun to be elucidated (Fuentemilla, Penny, Cashdollar, Bunzeck, & Düzel, 2010). Speculatively, it could be argued that the hippocampus, a brain region considered to be important in binding information together, could provide the necessary neural code along which items, and their features, can be temporally structured and accessed (Jensen & Lisman, 2005). Further studies will need to be conducted to confirm these suspicions.

### 4.4 Summary

This study sought to examine the differential effect that having to ignore irrelevant information or update information had on overall WM recall and how the mental representations that support this behaviour vary according to feature similarity between relevant and irrelevant information. Ignoring, even after having accounted for time, was found to have the most detrimental effect on overall recall and to be associated with the highest level of misbinding – erroneously combining features together in memory. These results reveal that how irrelevant information affects WM recall varies greatly according to the prior relevance of that information (Fallon, et al., 2016) and that feature overlap between relevant and irrelevant information, though subtle, can affect the underlying mental representations used to support these behaviours.

## Appendix A Supplementary data

mmc1
