# Supplementary material for: Ignoring versus updating in working memory reveal differential roles of attention and feature binding
Source: Cortex. 2018 Oct;107:50–63. doi: 10.1016/j.cortex.2017.12.016 (PMC6181802; doi:10.1016/j.cortex.2017.12.016)
Supplement: mmc1 [file mmc1.pdf]

Supplementary Materials

	Location	
Colour	***Congruent***	***Incongruent***
Congruent	n= 21	n =21
Incongruent	n =22	n = 19

Supplemental Table 1: Number of participants in each condition.

Modelling parameters

Kappa

Kappa is an index of how concentrated (width) a response is around the presented memoranda on a trial. A higher kappa is indicative of a higher concentration around an item's orientation and is therefore considered to reflect good performance.

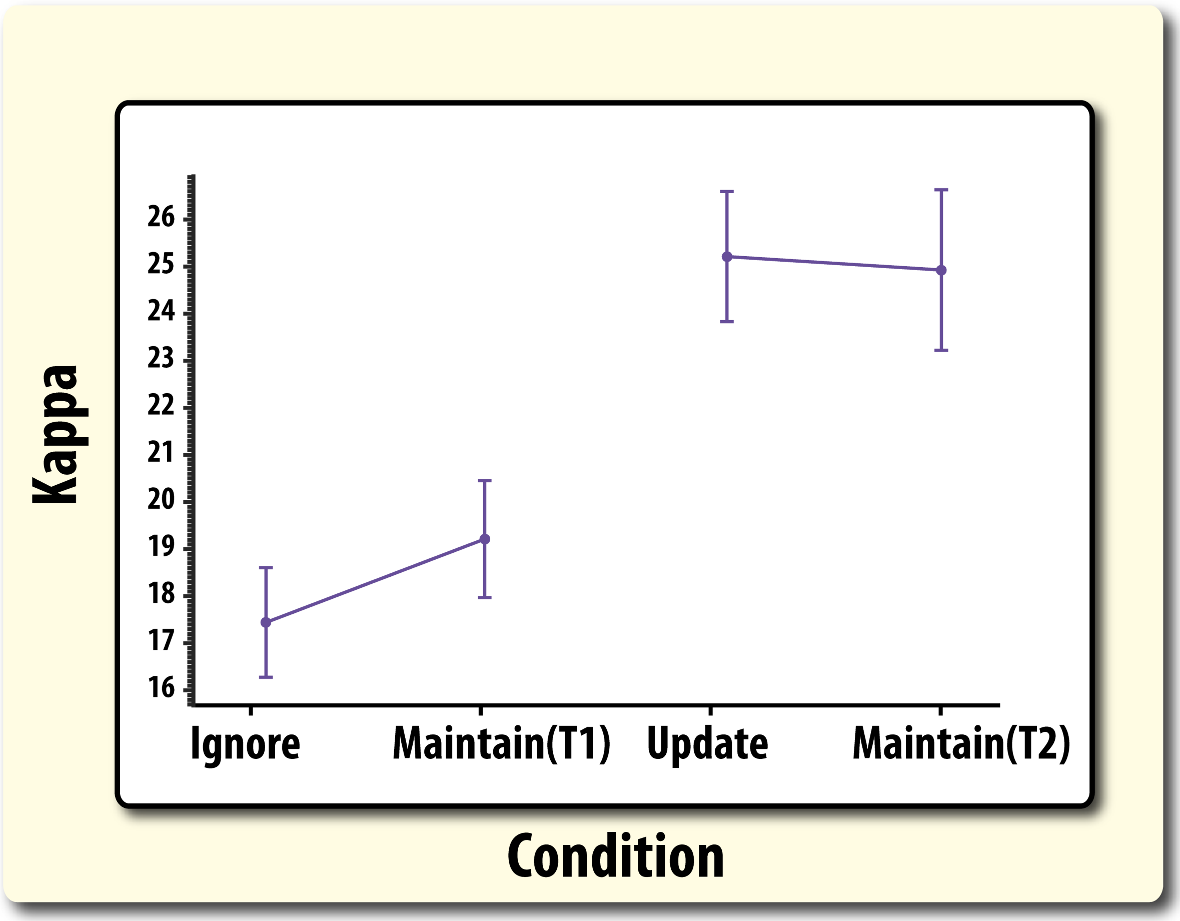

Supplementary Figure 1: Retention period affected kappa – variability in recall of target items. Error bars reflect standard error of the mean (SEM).

No significant main effects of colour ($F(1,79) = 2.70$, $p = .10$) or location ($F(1,79) = 3.10$, $p = .082$) were observed. However, there was a significant main effect of retention period ($F(1,79) = 84.55$, $p < .0001$; **Supplementary Figure 1**), whereby shorter delays between target and probe were associated with a higher kappa than longer durations. This effect was found to significantly vary with colour congruency ($F(1,79) = 4.01$, $p = .049$). This significant effect was due to the effect of time (maintenance period) on kappa values being accentuated in the colour congruent condition ($t(79) = 7.97$, $p < .0001$) compared to the incongruent condition ($t(79) = 5.05$, $p < .0001$). That is, information was seen to decay to a greater extent with time in those conditions were the two frames contained the same colour values, compared to when they contained separate colour values.

Kappa values did not significantly vary according to the presence or absence of irrelevant information (F(1,79) = 1.27, p = .26), but the effect of this manipulation significantly varied between the location congruency groups (F(1,79) = 4.11, p = .046; **Supplementary Figure 2**). Decomposing this interaction, the presence of irrelevant information was only found to significantly reduce kappa when the target and irrelevant information shared the same spatial locations (t(79) = 2.27, p = .026), but not when the locations differed ($t<1$). Thus, there was a direct effect of location congruency on the effect irrelevant information had on the fidelity of mental representations.

There also appears to be evidence of an indirect effect of location congruency; significantly higher kappa values were observed on the maintain only trials when the locations were the same, compared to when they were not the same (t(79) = 2.05, p = .043). Thus, recall of memoranda was more precise when the locations were the same for the target and irrelevant information compared to when they were different, even though no irrelevant information was presented on these trials. There was no such effect in the trials that contained irrelevant information (t(79) = 1.31, p = .194).

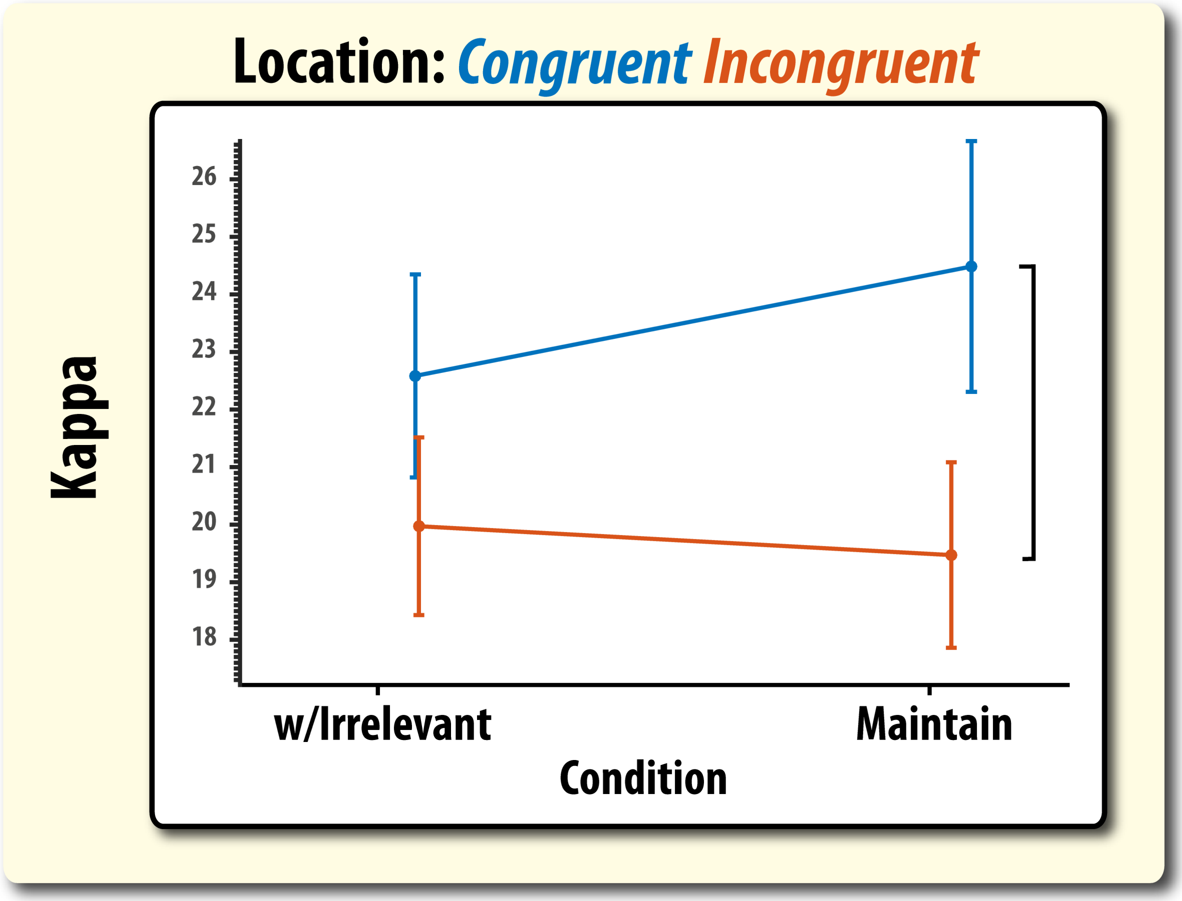

Supplementary Figure 2: Kappa as a function of task (maintain only or deal with irrelevant information (ignore and update). Error bars reflect standard error of the mean (SEM).

None of the other interactions were significant (*p values*>.27). Thus, cumulatively, two notable effects of colour and spatial congruence were observed on kappa. Colour congruency modulated the effect of time on the decrease in the fidelity of information over time. When the colours used for target and irrelevant items was the same, compared to when they were different, there was a greater decline in kappa across time. Though, this effect was not specific to the presence of irrelevant information. Spatial congruence was also found to determine the effect irrelevant information had on kappa: irrelevant information only reduced the fidelity of mental representations when the target and irrelevant information appeared in the same spatial locations. This effect seemed to be driven by an *increase* in kappa for the maintain only trials.

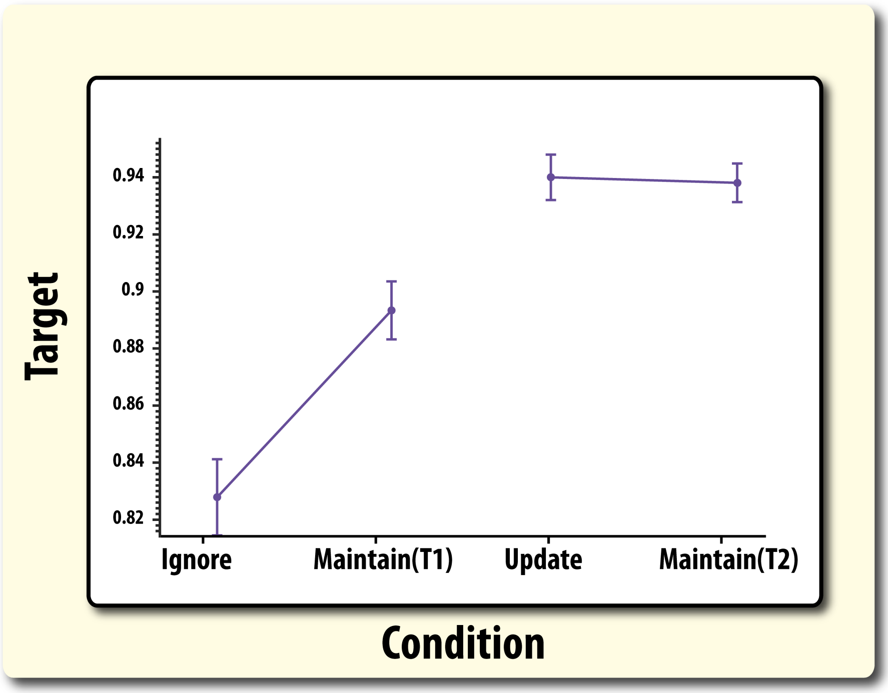

Supplementary Figure 3: Probability of responding to the target orientation according to task. Error bars reflect standard error of the mean (SEM).

Target response

The probability of responding to the target item was found to be significantly reduced by longer retention periods ($F(1,79) = 90.07$, $p < .0001$; **Supplementary Figure 3**) and was also significantly reduced by the presence of irrelevant information ($F(1,79) = 18.97$, $p < .0001$). These two factors significantly interacted ($F(1,79) = 27.75$, $p < .0001$), an effect due to the probability of responding to the target being reduced on ignore trials compared to its temporal control ($t(79) = 6.09$, $p < .0001$), whereas this was not the case when comparing the update condition with its temporal control ($t(79) = .37$, $p = .71$). There was no significant main effect of spatial congruence ($F(1,79) = 2.47$, $p = .119$). None of the other effects were significant (p's $> .211$). In summary, having to ignore irrelevant distracters leads to a significant decrease in the probability of responding to the target item, even

when accounting for the effect of retention period. This did not seem to vary according to the spatial or colour congruence of targets and distracters.

Guessing

Longer retention periods led to a significant increase in the number of guess responses ($F(1,79) = 68.34$, $p < .0001$). Despite the lack of a significant effect for introducing irrelevant information ($F<1$), there was a significant interaction between retention period and presence of irrelevant information ($F(1,79) = 4.85$, $p = .031$), indicating a differential effect on ignoring and updating. This was due to there being less guess responses in the updating condition compared to its temporal control ($t(79) = 3.00$, $p = .005$), but there was no significant difference between the ignore condition and its temporal control ($t(79) = 1.09$, $p = .277$). There was a trend towards a significant interaction between the presence of irrelevant information and colour congruence ($F(1,79) = 3.23$, $p = .076$). None of the effects were significant (all p's > .256).

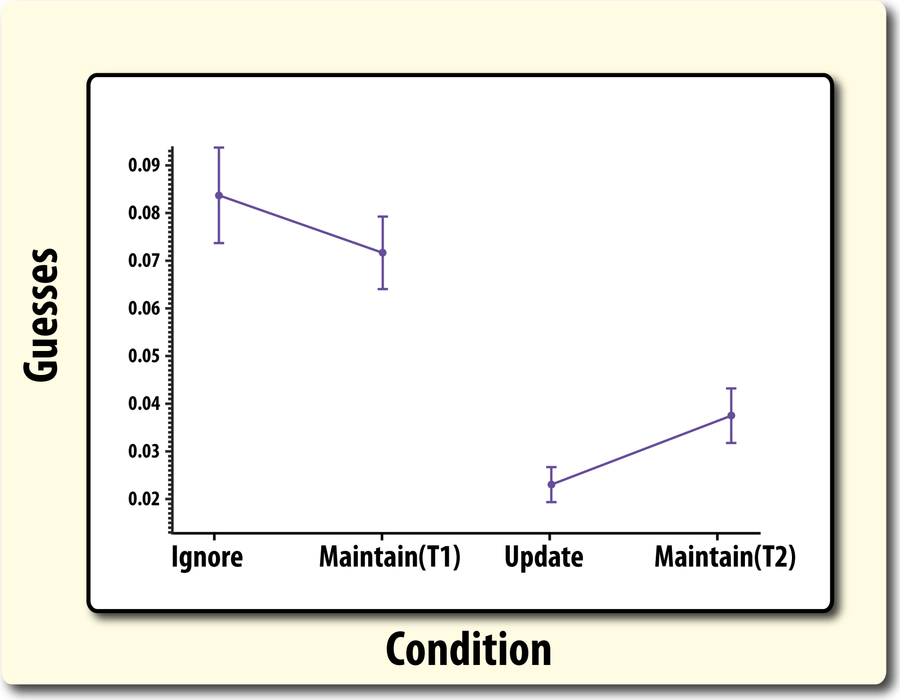

Supplementary Figure 4: Guess responses according to task. Error bars reflect standard error of the mean (SEM).

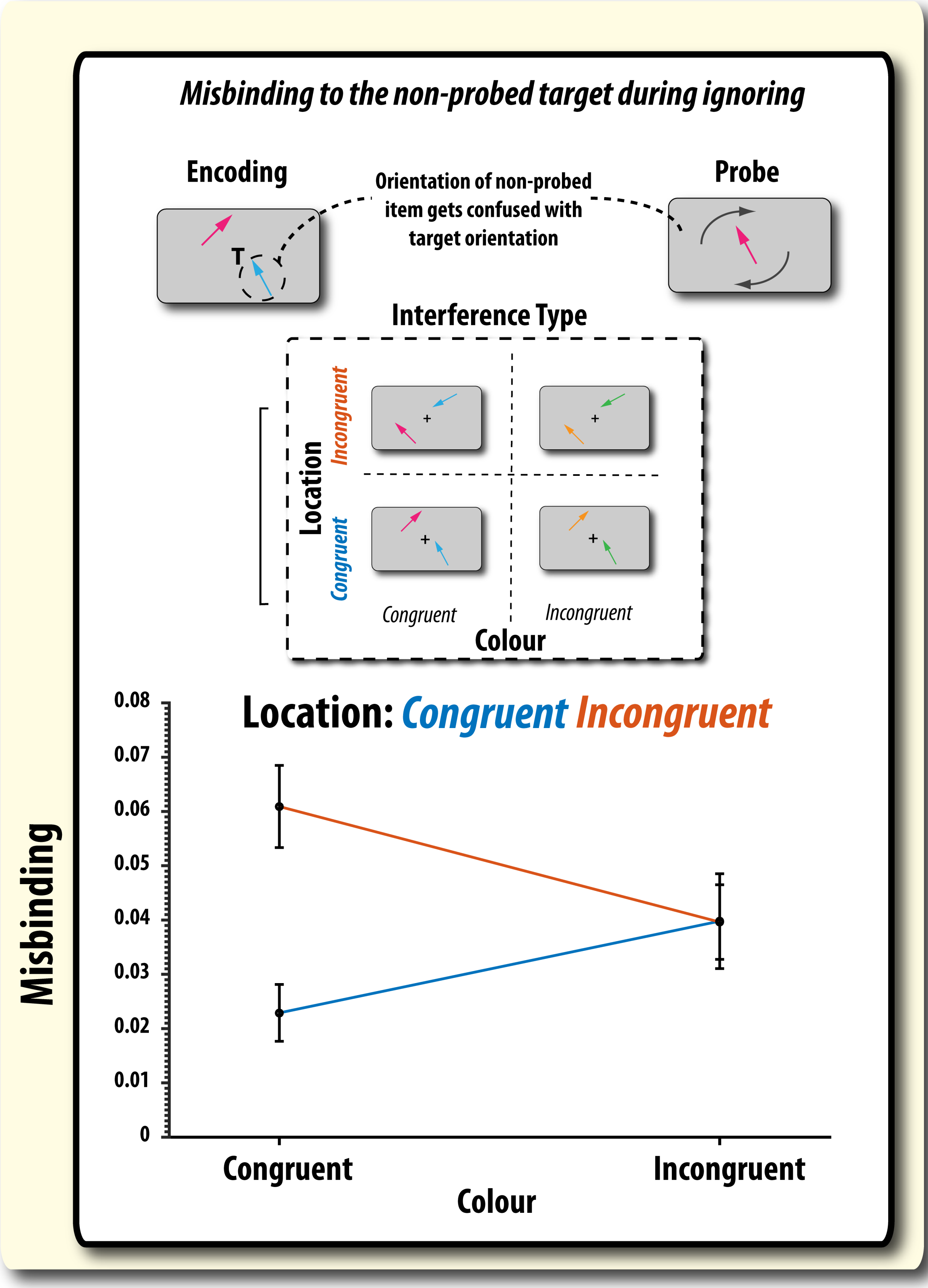

Supplemental Figure 5. Misbinding to the non-probed target in the ignore condition according to feature similarity. Note, that this is the same data from the left most bars in each panel of Figure 7 (A-D), but plotted in a different fashion. Error bars reflect standard error of the mean (SEM).

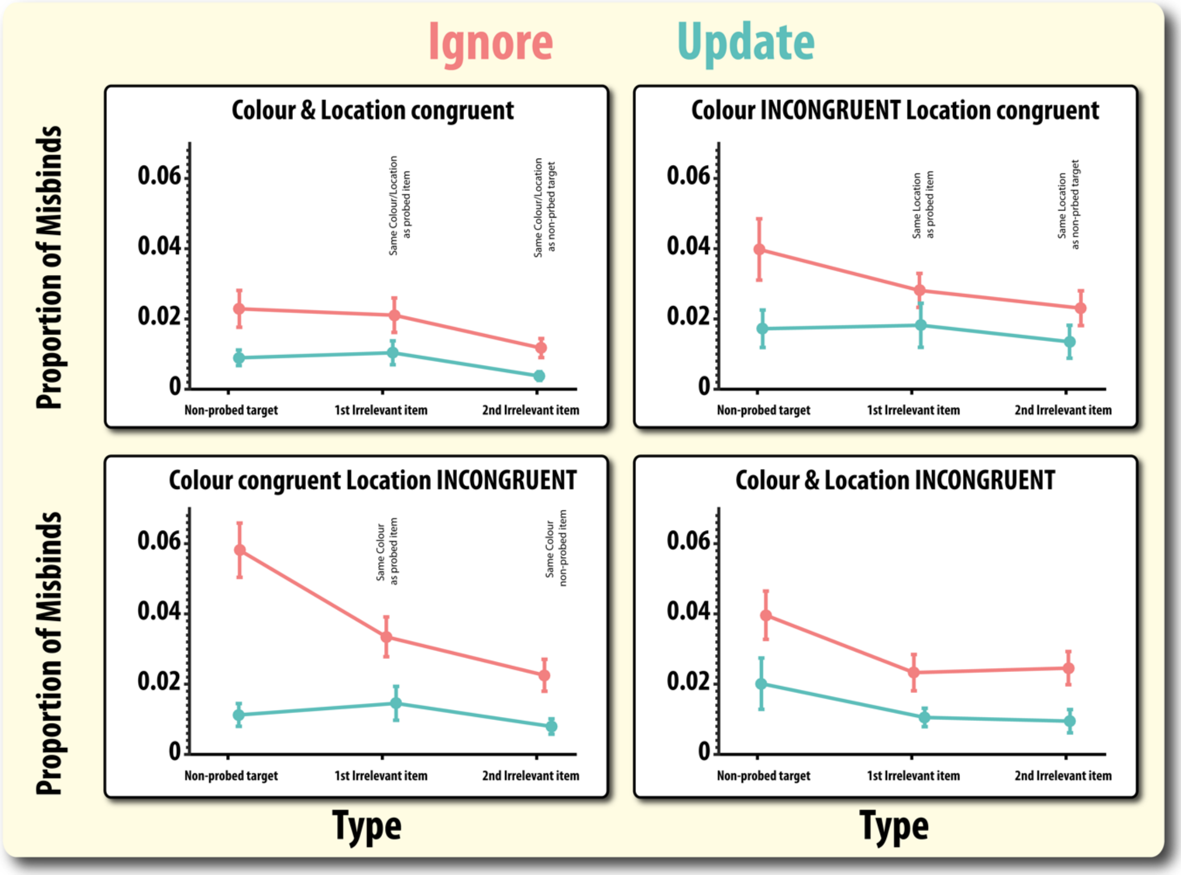

Supplementary Figure 6 | Levels of misbinding for ignore and update conditions across each feature congruence condition. Note, given that both irrelevant items were presented in different colours and at different locations, each item was randomly assigned to being either irrelevant item 1 or irrelevant item 2. Error bars reflect standard error of the mean (SEM).

Model Evaluation

A similar model validation procedure was done as in previous studies (Fallon et al, 2016; Fallon et al, 2017); we compared the model fits (Akaike Information Criterion; AIC) with and without the salient parameters in the model (misbinding and guessing).

Separately for each feature congruence condition, but across all four tasks (ignore, maintain (t1), update and maintain (t2), we examined the AIC for three models: full model (all parameters), full model without the misbinding parameter and full model without the guessing parameter). The full model was found to have a better (lower) AIC for all four feature congruence conditions.

Colour	Location	Full model	Full model minus misbinding parameter	Full model minus guessing parameter
Congruent	Congruent	2311	2325	3064
Incongruent	Congruent	3968	4078	4482
Congruent	Incongruent	3909	4100	4535
Incongruent	Incongruent	4820	4901	5539

Supplementary Table 2 Model fits (AIC values) across each feature congruence condition
